# Commentary: Activation of Pedunculopontine Glutamate Neurons Is Reinforcing

**DOI:** 10.3389/fnbeh.2017.00150

**Published:** 2017-08-08

**Authors:** Mahsa Mayeli, Farzaneh Rahmani

**Affiliations:** ^1^Student's Scientific Research Center (SSRC), Tehran University of Medical Sciences Tehran, Iran; ^2^Neuroimaging Network (NIN), Universal Scientific Education and Research Network (USERN) Tehran, Iran

**Keywords:** optogenetics, ventral tegmental area, pedunculopontine tegmental nucleus, glutamate, dopamine

## Ventral tegmental area re-evaluated

Midbrain Ventral Tegmental Area (VTA) has long been known for its pivotal role in mammalian reward system. A growing body of literature is now focusing on identification of the dominant element modulating neural populations of VTA, leading to Operant conditioning, a well-known mechanism by which a reward following a given stimulus can underscore a specific behavior, commonly termed as reinforcement learning.

We focus on the recent findings from Yoo et al. (2017), who implemented optogenetic method as a strong and trending tool for detection of specific circuits and cell dominancy of the VTA reward pathway. By cell-type selective approaches, they claim that glutamatergic projections of the pedunculopontine tegmental nucleus (PPTg) neurons to VTA, are the most important neural substrate of positive reinforcement among heterogonous population of dopaminergic, glutamatergic and GABAergic neurons of VTA.

To identify the presumed glutamate neurons projections to the VTA, they sterotactically infused an Adeno-associated viral vector expressing Channelrhodopsin-2 (ChR2) fused with a red fluorescent reporter. After pretest control measures addressing whether the target identifier was properly injected into dopaminergic regions in the PPTg, they put mice whose brain's VTA areas were inserted with fluorescent ChR2 and those inserted with fluorescent marker alone, for behavioral measurements on the nose poke assay and two bottle tests (Rizzi et al., 2016). Utilizing cell-attached recordings, high frequency optogenetic stimulation of PPTg glutamate terminals significantly increased VTA neurons firing rates. Although fast excitatory post synaptic currents (EPSCs) were detected in both dopamine and non-dopamine neurons, EPSC was significantly higher in dopamine neurons. Mice bearing ChR2 coupled with red fluorescent receptor in their PPTg-VTA system were able to develop a certain preference for nose poke holes that provided photostimulation, made more active nose pokes compared to mice injected with red fluorescent alone, and learned to actively lick more water from sippers coupled with laser stimulation, when compared to controls. The study suggests that glutamatergic inputs from PPTg to VTA preferentially target dopaminergic populations and can serve as a positive reinforcer.

## Optogenetics enlighten the way

VTA encompasses glutamatergic, GABAergic and dopaminergic neurons, while receiving glutamatergic as well as cholinergic projections from PPTg and a wide array of other cortical and subcortical areas (Geisler et al., 2007). Indeed convergence of glutamatergic afferents, from prefrontal cortical areas to mesopontine PPTg nuclei is mandatory for the integrative and deterministic activity of VTA in the reward system (Geisler et al., 2007).

Optogenetic methods have formerly provided sufficient evidence that certain projections from dorsal raphe (DR) increase dopamine release in VTA (Qi et al., 2014). In the mentioned study mice actively learnt a wheel-turning response to receive photostimulation. While previous reports had emphasized on the serotonergic input of DR nucleus to the VTA (Olds and Milner, 1954), Qi et al. emphasize on dopaminergic input to the VTA from DR and further pursuit vesicular glutamate transporter 3 dopaminergic input to the mesoaccumbens nucleus. Relative contribution level of different inputs to the final output from VTA remains to be validated.

On the contrary, via optogenetic photoinhibition, Yau and his colleagues demonstrated that both cholinergic and glutamatergic projections from PPTg to VTA are mandatory for operant conditioning, further supporting an indisputable role for VTA non dopamine neurons in behavioral reinforcement (Yau et al., 2016).

Regarding the output projections, Morales et al. suggested that stimulation of GABAergic and glutamatergic subpopulations of the PPTg neurons is capable of evoking reward system to produce an identical activation of motivated behavior in mice stimulated in these areas (Morales and Margolis, 2017). Interestingly, an increase in GABA neurons' firing rate in was detected in response to noxious stimuli, i.e., footshock (Barage and Sonawane, 2015), and photoactivation of glutamatergic projections of the VTA to nucleus accumbens drived aversion in another study (Qi et al., 2016). These indicate possible deterministic role for the recently found combinatorial neurons, capable of co-releasing dopamine and glutamate, of the VTA, that can lead to goal-directed behavior. The underpinnings of the release-patterns associated with these combinatorial neurons is just beginning to be understood and is currently being considered a focus of intense research endeavors (Morales and Margolis, 2017). This simultaneous release of multiple neurotransmitters by the same neuron can enhance the spatial and temporal control of synaptic transmission. Molecular evidence also support co-expression of Choline acetyltransferase and GABA in PPTg cultures, putting further spin on the role of combined neurotransmitter release (Pienaar et al., 2016).

This newly found controversial insight might revolutionize the former approaches addressing this issue, including this study by Yoo et al. considering the fact that the experiment determined the increased dopamine as the sole marker of activation of dopamine cells, there is a possibility that this increase was due to activated dopamine-glutamate combinatorial neural populations. This hypothesis seems probable as in former studies, focusing on one specific neurotransmitter (glutamate or dopamine) to activate the reward pathway, possible simultaneous increase in the other neurotransmitter was not measured. Besides, both dopamine and glutamate release were shown to mediate activation of the reward system. Moreover, the study mainly focuses on the dominancy of afferent glutamate projections to the VTA, however, taking these combinatorial neural populations into account, these afferent projections might essentially activate glutamate-dopamine populations, instead of mere dopamine neurons, therefore, activating the reinforcement learning through both dopamine and glutamate release.

In conclusion, although a great number of former studies has emphasized on the dominancy of one specific neurotransmitter underling operant conditioning process, accumulated evidence considering these combinatorial neural populations is now suggesting that further illustrations on compartmentalization of neural populations of VTA and an evaluation on their level of involvement in reinforcement-based behavior is required.

## Author contributions

MM drafted the manuscript and proposed the idea. FR made critical review of the subject and helped drafting the manuscript.

### Conflict of interest statement

The authors declare that the research was conducted in the absence of any commercial or financial relationships that could be construed as a potential conflict of interest. The reviewer AJR and handling Editor declared their shared affiliation, and the handling Editor states that the process nevertheless met the standards of a fair and objective review.

## References

[B1] Barage S. H. Sonawane K. D. (2015). Amyloid cascade hypothesis: pathogenesis and therapeutic strategies in Alzheimer's disease. Neuropeptides 52, 1–18. 10.1016/j.npep.2015.06.008 26149638

[B2] Geisler S. Derst C. Veh R. W. Zahm D. S. (2007). Glutamatergic afferents of the ventral tegmental area in the rat. J. Neurosci. 27, 5730–5743. 10.1523/jneurosci.0012-07.2007 17522317 PMC3202987

[B3] Morales M. Margolis E. B. (2017). Ventral tegmental area: cellular heterogeneity, connectivity and behaviour. Nat. Rev. Neurosci. 18, 73–85. 10.1038/nrn.2016.165 28053327

[B4] Olds J. Milner P. (1954). Positive reinforcement produced by electrical stimulation of septal area and other regions of rat brain. J. Comp. Physiol. Psychol. 47, 419–427. 13233369 10.1037/h0058775

[B5] Pienaar I. S. Vernon A. Winn P. (2016). The cellular diversity of the pedunculopontine nucleus: relevance to behavior in health and aspects of Parkinson's disease. Neuroscientist 23, 415–431. 10.1177/1073858416682471 27932591

[B6] Qi J. Zhang S. Wang H. L. Barker D. J. Miranda-Barrientos J. Morales M. (2016). VTA glutamatergic inputs to nucleus accumbens drive aversion by acting on GABAergic interneurons. Nat. Neurosci. 19, 725–733. 10.1038/nn.4281 27019014 PMC4846550

[B7] Qi J. Zhang S. Wang H.-L. Wang H. de Jesus Aceves Buendia J. Hoffman A. F.. (2014). A glutamatergic reward input from the dorsal raphe to ventral tegmental area dopamine neurons. Nat. Commun. 5, 5390–5390. 10.1038/ncomms6390 25388237 PMC4231541

[B8] Rizzi G. Lodge M. E. Tan K. R. (2016). Design and construction of a low-cost nose poke system for rodents. MethodsX 3, 326–332. 10.1016/j.mex.2016.04.002 27222822 PMC4855068

[B9] Yau H. J. Wang D. V. Tsou J. H. Chuang Y. F. Chen B. T. Deisseroth K.. (2016). Pontomesencephalic tegmental afferents to VTA non-dopamine neurons are necessary for appetitive pavlovian learning. Cell Rep. 16, 2699–2710. 10.1016/j.celrep.2016.08.007 27568569 PMC6324191

[B10] Yoo J. H. Zell V. Wu J. Punta C. Ramajayam N. Shen X.. (2017). Activation of pedunculopontine glutamate neurons is reinforcing. J. Neurosci. 37, 38–46. 10.1523/jneurosci.3082-16.2017 28053028 PMC5214635

